# Screening for Systemic Diseases Associated with Dental Self-Care in Japanese Adolescents

**DOI:** 10.3390/jcm13206087

**Published:** 2024-10-12

**Authors:** Masanobu Abe, Akihisa Mitani, Kazuto Hoshi, Shintaro Yanagimoto

**Affiliations:** 1 Division for Health Service Promotion, University of Tokyo, Tokyo 113-0033, Japan; mitania-int@h.u-tokyo.ac.jp (A.M.); yanagimoto@hc.u-tokyo.ac.jp (S.Y.); 2 Department of Oral & Maxillofacial Surgery, University of Tokyo Hospital, 7-3-1 Hongo, Bunkyo-ku, Tokyo 113-8655, Japan; hoshi-ora@h.u-tokyo.ac.jp

**Keywords:** toothbrushing, oral health, oral hygiene, periodontal disease, gingivitis, periodontitis

## Abstract

**Background:** Toothbrushing is important for maintaining oral health and preventing periodontal disease. However, the association between toothbrushing and systemic diseases remains unclear in adolescence. In this study, the association between dental self-care (frequency and duration of toothbrushing) and systemic diseases/disorders in adolescents was examined. **Methods:** We conducted a retrospective review of mandatory medical questionnaires administered during legally mandated freshman medical checkups between 2017 and 2019 at the University of Tokyo, Japan. Out of 9376 total responses, 9098 cases involving individuals under the age of 20 were included in the analysis. Respondents were classified into three groups based on their daily toothbrushing frequency: “1 time or less”, “twice”, and “3 times or more”. For the duration of each toothbrushing session, they were classified into three groups: “1 min or less”, “2–3 min”, and “4 min or more”. A statistical analysis was performed by Pearson’s *χ*^2^ test and multinomial logistic regression analysis. **Results:** Regarding frequency of daily toothbrushing: The *χ*^2^ test showed no significant relationship between frequency of toothbrushing and 17 systemic diseases/disorders. A multivariate analysis found that gingival bleeding and sex were independent factors. The risk of gingival bleeding decreased dramatically with increased frequency of toothbrushing (odds ratio (OR): 0.428; 95% confidence interval (CI), 0.366–0.501; *p* < 0.001). Regarding the amount of time spent on toothbrushing: The *χ*^2^ test showed atopic dermatitis and arrhythmia were significantly associated with the duration of toothbrushing (*p* = 0.032 and *p* = 0.016, respectively). In the multivariate analysis, atopic dermatitis, gingival bleeding, and sex were independent factors regarding the duration of toothbrushing; longer brushing time was associated with a lower risk of atopic dermatitis (OR: 0.731, 95% CI: 0.578–0.924, *p* = 0.009) and a lower risk of gingival bleeding (OR: 0.643, 95% CI: 0.567–0.729, *p* < 0.001). **Conclusions:** Dental self-care was most strongly associated with gingival bleeding, while the risk of atopic dermatitis was found to increase with shorter toothbrushing times. The results suggest that dental self-care during adolescence is important not only for oral health but also for general health.

## 1. Introduction

Oral diseases are known to be of critical public health importance due to their high prevalence. In particular, dental caries and periodontal disease are among the most prevalent diseases in the world [1,2]. People who brush their teeth less frequently are at higher risk of developing dental caries and periodontal disease [3,4,5]. Furthermore, it has been reported that oral hygiene care, including toothbrushing, is involved not only in oral diseases but also in systemic diseases such as cardiovascular disease, cerebrovascular disease, hypertension, metabolic syndrome, diabetes, dyslipidemia, and chronic kidney disease in adults [6,7,8,9,10,11,12,13,14,15,16]. Despite these known associations, the direct relationship between toothbrushing practices and systemic diseases in adolescents remains underexplored. We have previously shown that the frequency and duration of daily toothbrushing in adolescents are closely associated with gingival bleeding [5]. Although toothbrushing twice a day is generally recommended [17], it was shown that brushing three or more times a day is preferable in terms of preventing gingivitis [5]. Furthermore, although the duration of toothbrushing has not been discussed much, it was shown that more than four minutes per brushing session is desirable in order to prevent gingivitis [5]. It has recently been shown that gingival bleeding is closely associated with systemic diseases that are common in adolescents [18]. However, the relationship between systemic diseases in adolescents and toothbrushing enrichment is not elucidated. Therefore, in this study, we investigated the relationship between dental self-care (frequency of daily toothbrushing and duration of toothbrushing per time) and systemic diseases in Japanese adolescents.

## 2. Methods

### 2.1. Population and Study Design

We retrospectively analyzed data from 9376 medical questionnaires during new student medical examinations conducted at the University of Tokyo from April 2017 to April 2019. The medical questionnaire was distributed to all new students during the specified time period, and its submission was a mandatory part of the new student health examination. The questionnaire was self-administered and consisted of closed and open-ended questions, as described in our previous report [5,18].

Of the 9376 total response sets, all 9098 cases under the age of 20 (17–19 years; mean age 18.3 years) were examined for the relationship between frequency/duration of tooth brushing and systemic diseases/disorders. Of the 9098 cases under the age of 20, data from 9072 to 9070 respondents were valid for frequency of daily brushing and the duration of toothbrushing per time, respectively. According to the frequency of daily brushing, the respondents were classified into three groups as in the previous report: “1 time or less”, “twice”, and “3 times or more”; for the duration of toothbrushing, respondents were also classified into three groups as in the previous report: “1 min or less”, “2–3 min”, and “4 min or more” [5]. Systemic diseases/disorders with more than 50 cases of morbidity were included in this study. The systemic diseases/disorders were as follows: pollinosis, food and drug allergy, inhaled antigen allergy except pollinosis, allergic rhinitis, asthma/cough variant asthma, otitis media/externa, sinusitis, pneumothorax/mediastinal emphysema, atopic dermatitis, urticaria, scoliosis, spondylosis/spondylolisthesis/hernia, strabismus, myopia/hyperopia/astigmatism, arrhythmia, abnormal electrocardiographic (ECG) other than arrhythmia, anemia [19,20]. Gingival bleeding and sex were added to explanatory variables in the multivariate analysis.

### 2.2. Statistical Analysis

Statistical analysis was performed using Pearson’s *χ*^2^ test and multinomial logistic regression analysis. A value of *p* < 0.05 (two-sided) was determined as significant. All the analyses were performed using the statistical software program SAS ver. 9.4 (SAS Institute Inc., Cary, NC, USA) and SPSS Statistics 25 (IBM, Armonk, NY, USA) for the analyses.

### 2.3. Institutional Approval

This retrospective study was approved by the research ethics committee of the University of Tokyo on 14 September 2018, approval no. 18-197 (revised to No. 24-102 on 29 May 2024).

## 3. Results

### 3.1. Association between the Frequency of Daily Toothbrushing and Systemic Diseases/Disorders

Of the data of 9098 students aged 17–19, that from 9072 students were valid for the frequency of toothbrushing. Students were categorized into three groups by the toothbrushing frequency; “1 time or less (*n* = 1868)”, “twice (*n* = 5971)”, and “3 times or more (*n* = 1233)” [5]. The associations between the frequency of daily toothbrushing and systemic diseases/disorders in adolescence were examined by Pearson’s *χ*^2^ tests and multinomial logistic regression analysis.

In Pearson’s *χ*^2^ tests, the associations between frequency of daily toothbrushing and 17 systemic diseases/disorders (pollinosis, food/drug allergy, inhaled antigen allergy, allergic rhinitis, otitis media/externa, sinusitis, pneumothorax/mediastinal emphysema, asthma/cough-variant asthma, atopic dermatitis, urticaria, scoliosis, spondylosis/spondylolisthesis/hernia, strabismus, myopia/hyperopia/astigmatism, arrhythmia, abnormal ECG other than arrhythmia, anemia) were examined. The *χ*^2^ test showed no significant relationship between the frequency of toothbrushing and 17 systemic diseases/disorders (Table 1).

The multivariate analysis using a multinomial logistic regression model with toothbrushing frequency as the objective variable and the 17 systemic diseases/disorders plus gingival bleeding (an oral disorder) and sex for a total of 19 items as explanatory variables revealed significant rates of the following: gingival bleeding (odds ratio (OR): 0.428, 95% confidence interval (CI): 0.366–0.501, *p* < 0.001), sex female (OR: 3.167, 95% CI: 2.605–3.849, *p* < 0.001) (Table 2). The result indicated that the risk of gingival bleeding decreased with toothbrushing frequency, and females brushed their teeth more frequently than males.

### 3.2. Association between the Duration of Toothbrushing per Time and Systemic Diseases/Disorders

Of the data of 9098 students, that from 9070 students were valid for the duration of toothbrushing per time. The 9070 students were categorized into three groups by the duration of toothbrushing per time; “1 min or less (*n* = 1508)”, “2–3 min (*n* = 4291)”, and “4 min or more (*n* = 3271)” [5]. The associations between the duration of toothbrushing and systemic diseases/disorders in adolescence were analyzed by Pearson’s *χ*^2^ tests and multinomial logistic regression analysis.

In Pearson’s *χ*^2^ tests, the associations between the duration of toothbrushing and 17 systemic diseases/disorders were examined. The *χ*^2^ test showed that atopic dermatitis and arrhythmia were significantly associated with the duration of toothbrushing (*p* = 0.032 and *p* = 0.016, respectively) (Table 3).

The multivariate analysis using a multinomial logistic regression model with toothbrushing time as the objective variable and the 17 systemic diseases/disorders plus gingival bleeding (an oral disorder) and sex for a total of 19 items as explanatory variables revealed significant rates of following: atopic dermatitis (OR: 0.731, 95% CI: 0.578–0.924, *p* = 0.009), gingival bleeding (OR: 0.643, 95% CI: 0.567–0.729, *p* < 0.001), and sex female (OR: 1.203; 95% CI, 1.022–1.415; *p* = 0.026) (Table 4). The results showed that the risk of atopic dermatitis and gingival bleeding decreased with a longer duration of toothbrushing, with females brushing their teeth longer than males.

## 4. Discussion

The high prevalence of oral diseases is of critical public health importance, with dental caries and periodontal disease being among the ten most prevalent diseases in the world [1,2]. In particular, periodontal disease has been shown not only to cause tooth loss but also to be closely related to general health [21]. Periodontal disease has been linked to a range of diseases, including respiratory disease [22,23,24,25,26,27], cardiovascular diseases [28,29,30,31,32,33,34,35,36], diabetes mellitus [37,38], rheumatoid arthritis [39,40], metabolic syndrome [41], osteoporosis [42], preterm birth and fetal growth restriction [43,44], Alzheimer’s disease [45,46].

The association between periodontal disease and systemic diseases usually becomes apparent in middle age and later. However, one in three adolescents is reported to have symptoms of gingival bleeding, an early manifestation of periodontal disease, and it has recently been shown that gingival bleeding is closely associated with systemic diseases that are common in adolescents [18]. Therefore, prevention of periodontal disease from adolescence is considered important [47,48], and our previous studies have shown that dental self-care is key to gingivitis prevention: brushing teeth three times a day for at least four minutes each time was found to be effective in preventing gingival bleeding in adolescents [5]. However, the direct relationship between toothbrushing enrichment and systemic diseases in adolescents has not been clarified yet. Thus, this study investigated the relationship between dental self-care adequacy and systemic diseases among Japanese adolescents. The results showed that dental self-care was most strongly associated with gingival bleeding, while the risk of atopic dermatitis was found to increase with shorter toothbrushing time.

Atopic dermatitis is the most common eczematous inflammatory skin disease, with lesions typically presenting as erythematous and scaly papules and plaques without well-defined boundaries [49]. Although no reports on the relationship between toothbrushing and atopic dermatitis have been found as far as we have explored, a link between poor oral health and atopic dermatitis has been reported in recent years [50,51,52,53]. For example, Tan et al. showed new findings of a higher prevalence of dental abnormalities in patients with moderate to severe atopic dermatitis [51]. Macklis et al. examined the relationship between skin diseases and various oral health indicators and showed an association between atopic dermatitis and gingivitis, toothache, and oral infections [52]. Park et al. found that atopic dermatitis was significantly associated with the occurrence of dental caries in adults in Korea. Based on their findings, they note that dermatologists should be aware of dental symptoms in patients with atopic dermatitis and recommend regular dental checkups for early detection of dental caries [53]. In response to the emerging epidemiological evidence linking atopic dermatitis and poor oral health, some reports have explored the mechanisms involved [54,55]. Jiménez et al. found that atopic dermatitis and periodontitis independently had opposing effects on gingival crevicular fluid levels of IL-31, whereas atopic dermatitis alone affected levels of thymic stromal lymphopoietin [54]. Matsushima et al. found that mice with dermatitis show reduced salivary secretion and histological changes leading to periodontal disease and stated that proper control of skin inflammation is essential to prevent periodontal disease [55]. Research on atopic dermatitis and oral health has received a lot of attention in recent years, but there is still a lack of reports on the relationship between atopic dermatitis and toothbrushing.

On the other hand, evidence is accumulating for some diseases associated with oral hygiene behavior, and there are many reports on cardiovascular diseases [6,7,8]. A meta-analysis supports the hypothesis that more frequent toothbrushing decreases the risk of cardiovascular disease [6]. The results have important implications for cardiovascular disease prevention strategies. Furthermore, a nationwide population-based cohort study in South Korea showed that improved oral hygiene care was associated with a lower risk of developing atrial fibrillation and heart failure [7]. In the present study, in adolescents, the *χ*^2^ test also showed a significant relationship between shorter toothbrushing time and risk of arrhythmia. As arrhythmia was not an independent factor in the multivariate analysis, further research is needed on the relationship between toothbrushing and the risk of cardiovascular disease in adolescents. The relationship between oral hygiene behavior and blood pressure has also been reported in several studies; Del Pinto et al. reported that regular daily toothbrushing and electric toothbrushes were associated with improved blood pressure profiles [10]; Hwang et al. found that home oral hygiene was associated with blood pressure profiles and speculated that oral inflammation may contribute to the development of hypertension [11]. The relationship between oral hygiene behavior and cerebrovascular disease has also been reported in recent years. Chang et al. showed that infrequent oral hygiene care was positively associated with the risk of stroke. They state that brushing teeth three or more times a day may be associated with a lower risk of stroke [9]. The relationship between oral hygiene behavior and diabetes, dyslipidemia, and metabolic syndrome has also been reported. In a nationwide population-based cohort study, Chang et al. showed that improved oral hygiene through frequent tooth brushing reduced the risk of developing new diabetes [13]. Kuwabara et al. showed in a large Japanese cross-sectional study that the prevalence of diabetes and dyslipidemia was higher the lower the frequency of tooth brushing [14]. Kobayashi et al. found that the prevalence and incidence of metabolic syndrome were lower with more frequent toothbrushing. Their findings suggest the possibility of preventing metabolic syndrome via the inflammation/triglyceride pathway [12]. In recent years, the relationship between oral hygiene behavior and chronic kidney disease has also attracted attention. Chang et al. showed that frequent tooth brushing (more than three times a day) was negatively associated with the development of chronic kidney disease in a Korean national population-based retrospective cohort study [15]. Hirano et al. found that frequent toothbrushing is not only beneficial for oral health but may also be associated with a delay in the decline of renal function [16]. Their findings suggest that toothbrushing frequency may be a predictor of future decline in renal function.

The results of this study showed a significant relationship between toothbrushing time and atopic dermatitis in young people. This suggests that increasing the duration of toothbrushing may help improve atopic dermatitis. Of course, validation in other populations and longitudinal cohort studies are needed to confirm whether toothbrushing time is involved in improving atopic dermatitis. Furthermore, one of the most important findings of this study was that dental self-care was strongly associated with oral health (gingival bleeding). As periodontal disease is closely associated with various systemic diseases, especially in adults, it is considered important to build a good foundation of oral health through dental self-care during adolescence to prevent future periodontal disease.

## 5. Conclusions

Dental self-care was most strongly associated with gingival bleeding, while the risk of atopic dermatitis was found to increase with shorter toothbrushing times. The results suggest that dental self-care during adolescence is important not only for oral health but also for general health.

## Figures and Tables

**Table 1:** *χ*^2^ test for the relationship between frequency of daily toothbrushing and systemic diseases/disorders in Japanese adolescents.

			Frequency of Daily Toothbrushing				
			Time(s)				
Medical History			≤1	2	3≤	Total	*p*
All		*n* (%)	1868 (20.6)	5971 (65.8)	1233 (13.6)	9072 (100)	
Pollinosis	Absence	*n* (%)	1575 (20.6)	5037 (65.8)	1044 (13.6)	7656 (100)	0.957
	Presence		293 (20.7)	934 (66)	189 (13.3)	1416 (100)	
Food/Drug allergy	Absence	*n* (%)	1809 (20.6)	5791 (65.8)	1197 (13.6)	8797 (100)	0.923
	Presence		59 (21.5)	180 (65.5)	36 (13.1)	275 (100)	
Inhaled antigen allergy (except pollinosis)	Absence	*n* (%)	1827 (20.5)	5856 (65.9)	1208 (13.6)	8891 (100)	0.766
	Presence		41 (22.7)	115 (63.5)	25 (13.8)	181 (100)	
Allergic rhinitis	Absence	*n* (%)	1584 (20.6)	5070 (65.8)	1051 (13.6)	7705 (100)	0.942
	Presence		284 (20.8)	901 (65.9)	182 (13.3)	1367 (100)	
Otitis media/externa	Absence	*n* (%)	1823 (20.5)	5863 (65.9)	1208 (13.6)	8894 (100)	0.26
	Presence		45 (25.3)	108 (60.7)	25 (14.0)	178 (100)	
Sinusitis	Absence	*n* (%)	1832 (20.5)	5881 (65.9)	1214 (13.6)	8927 (100)	0.444
	Presence		36 (24.8)	90 (62.1)	19 (13.1)	145 (100)	
Pneumothorax/Mediastinal emphysema	Absence	*n* (%)	1844 (20.6)	5896 (65.8)	1221 (13.6)	8961 (100)	0.687
	Presence		24 (21.6)	75 (67.6)	12 (10.8)	111 (100)	
Asthma/Cough variant asthma	Absence	*n* (%)	1673 (20.4)	5412 (66)	1117 (13.6)	8202 (100)	0.376
	Presence		195 (22.4)	559 (64.3)	116 (13.3)	870 (100)	
Atopic dermatitis	Absence	*n* (%)	1722 (20.4)	5559 (65.9)	1152 (13.7)	8433 (100)	0.315
	Presence		146 (22.8)	412 (64.5)	81 (12.7)	639 (100)	
Urticaria	Absence	*n* (%)	1832 (20.5)	5897 (65.9)	1213 (13.6)	8942 (100)	0.077
	Presence		36 (27.7)	74 (56.9)	20 (15.4)	130 (100)	
Scoliosis	Absence	*n* (%)	1852 (20.6)	5933 (65.9)	1221 (13.6)	9006 (100)	0.342
	Presence		16 (24.2)	38 (57.6)	12 (18.2)	66 (100)	
Spondylosis/Spondylolisthesis/Hernia	Absence	*n* (%)	1860 (20.6)	5934 (65.8)	1224 (13.6)	9018 (100)	0.517
	Presence		8 (14.8)	37 (68.5)	9 (16.7)	54 (100)	
Strabismus	Absence	*n* (%)	1854 (20.6)	5924 (65.8)	1226 (13.6)	9004 (100)	0.718
	Presence		14 (20.6)	47 (69.1)	7 (10.3)	68 (100)	
Myopia/Hyperopia/Astigmatism	Absence	*n* (%)	1847 (20.5)	5923 (65.9)	1218 (13.6)	8988 (100)	0.234
	Presence		21 (25.0)	48 (57.1)	15 (17.9)	84 (100)	
Arrhythmia	Absence	*n* (%)	1851 (20.6)	5896 (65.8)	1218 (13.6)	8965 (100)	0.478
	Presence		17 (15.9)	75 (70.1)	15 (14.0)	107 (100)	
Abnormal ECG other than arrhythmia	Absence	*n* (%)	1854 (20.6)	5925 (65.8)	1224 (3.6)	9003 (100)	0.987
	Presence		14 (20.3)	46 (66.7)	9 (13.0)	69 (100)	
Anemia	Absence	*n* (%)	1861 (20.7)	5926 (65.8)	1224 (13.6)	9011 (100)	0.209
	Presence		7 (11.5)	45 (73.8)	9 (14.8)	61 (100)	

**Table 2:** Multinomial logistic regression analysis for the relationship between frequency of daily toothbrushing and systemic diseases/disorders in Japanese adolescents.

Medical History	OR (95% CI)	*p*
Pollinosis	0.983 (0.800–1.208)	0.871
Food/Drug allergy	1.008 (0.653–1.556)	0.972
Inhaled antigen allergy (except pollinosis)	0.952 (0.567–1.601)	0.853
Allergic rhinitis	1.049 (0.850–1.294)	0.656
Otitis media/externa	0.980 (0.591–1.624)	0.937
Sinusitis	0.838 (0.473–1.483)	0.543
Pneumothorax/Mediastinal emphysema	0.873 (0.432–1.766)	0.706
Asthma/Cough variant asthma	0.997 (0.774–1.284)	0.980
Atopic dermatitis	0.852 (0.635–1.144)	0.287
Urticaria	0.842 (0.478–1.486)	0.554
Scoliosis	0.741 (0.342–1.607)	0.448
Spondylosis/Spondylolisthesis/Hernia	1.831 (0.693–4.837)	0.222
Strabismus	0.720 (0.284–1.825)	0.489
Myopia/Hyperopia/Astigmatism	1.083 (0.545–2.153)	0.821
Arrhythmia	1.456 (0.715–2.962)	0.300
Abnormal ECG other than arrhythmia	1.151 (0.491–2.697)	0.746
Anemia	1.407 (0.513–3.861)	0.507
Sex (female)	3.167 (2.605–3.849)	<0.001 *
Gingival bleeding	0.428 (0.366–0.501)	<0.001 *

OR: odds ratio, CI: confidence interval, *: <0.05.

**Table 3:** *χ*^2^ test for the relationship between duration of toothbrushing (per time) and systemic diseases/disorders in Japanese adolescents.

			Duration of Toothbrushing				
			min				
Medical History			≤1	2–3	4≤	Total	*p*
All		*n* (%)	1508 (16.6)	4291 (47.3)	3271 (36.1)	9070 (100)	
Pollinosis	Absence	*n* (%)	1289 (16.8)	3611 (47.2)	2755 (36.0)	7655 (100)	0.448
	Presence		219 (15.5)	680 (48.1)	516 (36.5)	1415 (100)	
Food/Drug allergy	Absence	*n* (%)	1463 (16.6)	4170 (47.4)	3162 (36.0)	8795 ()	0.433
	Presence		45 (16.4)	121 (44)	109 (39.6)	275 (100)	
Inhaled antigen allergy (except pollinosis)	Absence	*n* (%)	1477 (16.6)	4219 (47.5)	3193 (35.9)	8889 (100)	0.092
	Presence		31 (17.1)	72 (39.8)	78 (43.1)	181 (100)	
Allergic rhinitis	Absence	*n* (%)	1272 (16.5)	3654 (47.4)	2777 (36.1)	7703 (100)	0.753
	Presence		236 (17.3)	637 (46.6)	494 (36.1)	1367 (100)	
Otitis media/externa	Absence	*n* (%)	1475 (16.6)	4219 (47.4)	3198 (36.0)	8892 (100)	0.179
	Presence		33 (18.5)	72 (40.4)	73 (41)	178 (100)	
Sinusitis	Absence	*n* (%)	1486 (16.6)	4225 (47.3)	3214 (36.0)	8925 (100)	0.698
	Presence		22 (15.2)	66 (45.5)	57 (39.3)	145 (100)	
Pneumothorax/Mediastinal emphysema	Absence	*n* (%)	1492 (16.7)	4238 (47.3)	3229 (36.0)	8959 (100)	0.805
	Presence		16 (14.4)	53 (47.7)	42 (37.8)	111 (100)	
Asthma/Cough variant asthma	Absence	*n* (%)	1366 (16.7)	3875 (47.3)	2959 (36.1)	8200 (100)	0.943
	Presence		142 (16.3)	416 (47.8)	312 (35.9)	870 (100)	
Atopic dermatitis	Absence	*n* (%)	1378 (16.3)	4000 (47.4)	3053 (36.2)	8431 (100)	0.032 *
	Presence		130 (20.3)	291 (45.5)	218 (34.1)	639 (100)	
Urticaria	Absence	*n* (%)	1483 (16.6)	4231 (47.3)	3226 (36.1)	8940 (100)	0.722
	Presence		25 (19.2)	60 (46.2)	45 (34.6)	130 (100)	
Scoliosis	Absence	*n* (%)	1501 (16.7)	4265 (47.4)	3238 (36.0)	9004 (100)	0.052
	Presence		7 (10.6)	26 (39.4)	33 (50.0)	66 (100)	
Spondylosis/Spondylolisthesis/Hernia	Absence	*n* (%)	1499 (16.6)	4266 (47.3)	3251 (36.1)	9016 (100)	0.987
	Presence		9 (16.7)	25 (46.3)	20 (37)	54 (100)	
Strabismus	Absence	*n* (%)	1501 (16.7)	4258 (47.3)	3243 (36.0)	9002 (100)	0.338
	Presence		7 (10.3)	33 (48.5)	28 (41.2)	68 (100)	
Myopia/Hyperopia/Astigmatism	Absence	*n* (%)	1493 (16.6)	4247 (47.3)	3246 (36.1)	8986 ()	0.479
	Presence		15 (17.9)	44 (52.4)	25 (29.8)	84 (100)	
Arrhythmia	Absence	*n* (%)	1482 (16.5)	4254 (47.5)	3227 (36.0)	8963 (100)	0.016 *
	Presence		26 (24.3)	37 (34.6)	44 (41.1)	107 (100)	
Abnormal ECG other than arrhythmia	Absence	*n* (%)	1499 (16.7)	4259 (47.3)	3243 (36.0)	9001 (100)	0.624
	Presence		9 (13.0)	32 (46.4)	28 (40.6)	69 (100)	
Anemia	Absence	*n* (%)	1497 (16.6)	4268 (47.4)	3244 (36.0)	9009 (100)	0.299
	Presence		11 (18.0)	23 (37.7)	27 (44.3)	61 (100)	

*: <0.05.

**Table 4:** Multinomial logistic regression analysis for the relationship between duration of toothbrushing (per time) and systemic diseases/disorders in Japanese adolescents.

Medical History	OR (95% CI)	*p*
Pollinosis	1.090 (0.914–1.299)	0.336
Food/Drug allergy	1.188 (0.829–1.704)	0.348
Inhaled antigen allergy (except pollinosis)	1.150 (0.747–1.769)	0.525
Allergic rhinitis	0.995 (0.836–1.184)	0.954
Otitis media/externa	1.105 (0.726–1.682)	0.64
Sinusitis	1.226 (0.744–2.021)	0.423
Pneumothorax/Mediastinal emphysema	1.285 (0.717–2.303)	0.399
Asthma/Cough variant asthma	1.074 (0.865–1.333)	0.520
Atopic dermatitis	0.731 (0.578–0.924)	0.009 *
Urticaria	0.778 (0.471–1.283)	0.325
Scoliosis	2.010 (0.882–4.579)	0.097
Spondylosis/Spondylolisthesis/Hernia	1.049 (0.474–2.321)	0.906
Strabismus	1.937 (0.840–4.470)	0.121
Myopia/Hyperopia/Astigmatism	0.736 (0.383–1.414)	0.358
Arrhythmia	0.793 (0.484–1.299)	0.357
Abnormal ECG other than arrhythmia	1.497 (0.701–3.196)	0.297
Anemia	1.037 (0.510–2.108)	0.920
Sex (female)	1.203 (1.022–1.415)	0.026 *
Gingival bleeding	0.643 (0.567–0.729)	<0.001 *

*: <0.05.

## Data Availability

Data are contained within the article.
